# Disease, Models, Variants and Altered Pathways—Journeying RGD Through the Magnifying Glass

**DOI:** 10.1016/j.csbj.2015.11.006

**Published:** 2015-11-26

**Authors:** Victoria Petri, G. Thomas Hayman, Marek Tutaj, Jennifer R. Smith, Stan Laulederkind, Shur-Jen Wang, Rajni Nigam, Jeff De Pons, Mary Shimoyama, Melinda R. Dwinell

**Affiliations:** Human and Molecular Genetics Center, Medical College of Wisconsin, USA; Department of Surgery, Medical College of Wisconsin, USA; Department of Physiology, Medical College of Wisconsin, USA

**Keywords:** Disease, Pathway, Variant, Animal model

## Abstract

Understanding the pathogenesis of disease is instrumental in delineating its progression mechanisms and for envisioning ways to counteract it. In the process, animal models represent invaluable tools for identifying disease-related loci and their genetic components. Amongst them, the laboratory rat is used extensively in the study of many conditions and disorders. The Rat Genome Database (RGD—http://rgd.mcw.edu) has been established to house rat genetic, genomic and phenotypic data. Since its inception, it has continually expanded the depth and breadth of its content. Currently, in addition to rat genes, QTLs and strains, RGD houses mouse and human genes and QTLs and offers pertinent associated data, acquired through manual literature curation and imported via pipelines. A collection of controlled vocabularies and ontologies is employed for the standardized extraction and provision of biological data. The vocabularies/ontologies allow the capture of disease and phenotype associations of rat strains and QTLs, as well as disease and pathway associations of rat, human and mouse genes. A suite of tools enables the retrieval, manipulation, viewing and analysis of data. Genes associated with particular conditions or with altered networks underlying disease pathways can be retrieved. Genetic variants in humans or in sequenced rat strains can be searched and compared. Lists of rat strains and species-specific genes and QTLs can be generated for selected ontology terms and then analyzed, downloaded or sent to other tools. From many entry points, data can be accessed and results retrieved. To illustrate, diabetes is used as a case study to initiate and embark upon an exploratory journey.

## Graphical abstract

**Figure:**
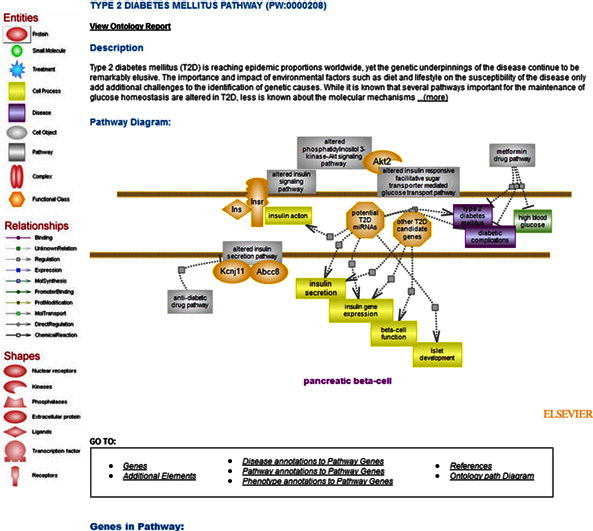

## 1 Introduction/Background

### 1.1 Diabetes

The prevalence of diabetes, despite continuous efforts geared at understanding the underlying mechanisms, the development of drugs and improved care management, continues to grow. The prognosis of its worldwide increase and the economic and healthcare burdens that accompany it, offer a somber picture. There are two major types of diabetes mellitus: type 1 diabetes mellitus (T1D) which results from the self-destruction of insulin producing pancreatic beta cells and represents ~ 10% of all cases and the more prevalent type 2 diabetes mellitus (T2D) of complex and still elusive etiology featuring both impaired insulin secretion and resistance/decreased sensitivity in target tissues. The defective insulin secretion, exhibited by both diabetes types albeit to varying degrees, affects glucose homeostasis. The hyperglycemic state—a hallmark of the condition, underlies a range of complications that affect the kidney, the eye, the cardiovascular systems and other organ systems. Genetic factors are likely involved but the quest for major culprit genes, particularly for T2D, has not been very fruitful. However, high-throughput experiments are broadening the range of potential players. Environmental factors such as diet and lifestyle are known to be important contributors. Evidence is accumulating for the impact of non-coding RNAs, epigenetics and the microbiota have in the complex network of diabetes etiology. A small percentage of diabetes is monogenic and is manifested early in life; defects affect the production/processing of insulin precursor. T2D, the major form of diabetes mellitus, once considered a condition of adult age, is increasingly affecting younger people [1], [2], [3], [4], [5], [6]. Pharmacologically, drugs that prompt insulin release by blocking the ATP-sensitive potassium channels, such as nateglinide and repaglinide or metformin, an antihyperglycemic drug, are among the agents that are currently used. Metformin mode of action is thought to involve the activation of adenosine monophosphate-activated kinase (AMPK) signaling pathway, an energy sensor and fuel regulator. However, the AMPK cascade is not the primary target; the mitochondrial respiratory chain complex I appears to be the main metformin target but the molecular mechanisms of its inhibition are not well understood. [7], [8], [9].

### 1.2 Balancing Glucose Levels: Insulin and Glucagon Secretion and Signaling

The pancreatic beta and alpha cells of the islets of Langerhans produce and release insulin and glucagon, respectively—two hormones essential for maintaining proper levels of glucose. When glucose levels are high, insulin is released to prompt glucose uptake in the muscle, heart and adipose tissues. Glucagon has the opposite effect: it promotes glucose release from liver storage by stimulating glycogenolysis and gluconeogenesis while inhibiting glycolysis and glycogenesis. Both hormones are expressed as precursors that need to be processed before they can engage their receptors. The mechanisms of glucagon release are incompletely understood. Once released, it can bind the glucagon receptor—a G-protein coupled receptor (GPCR) that activates the Galphas (G_s_) type of heterotrimeric G protein. The subsequent activation of adenylyl cyclase and production of cyclic AMP (cAMP) sets in motion the protein kinase A (PKA) signaling pathway. PKA signaling impacts a broad range of processes in a tissue- and cell-specific manner. In the context of glucagon, it underlies the glucagon-mediated effects on glucose levels. In contrast to glucagon, insulin secretion is a better understood regulatory network, especially the first phase of its biphasic release mode. When present, glucose is rapidly taken up by the beta cells and its oxidative metabolism leads to an increase in the ATP/ADP ratio which in turn triggers closure of ATP-sensitive potassium channels (K_ATP_) leading to membrane depolarization and opening of voltage-gated calcium channels (VDCC). The rise in calcium concentration promotes fusion of insulin-granules with the plasma membrane. The initial and rapid release phase involves granules at or near the plasma membrane. The second, slower but sustained release phase, is less well understood but it may involve factors resulting from pyruvate cycling in the course of glucose metabolism. Both phases of insulin secretion are impaired in T2D. Once released, insulin binds the insulin receptor—a receptor tyrosine kinase (RTK) that modulates a range of substrates to activate two downstream signaling cascades. The phosphatidylinositol 3-kinase-Akt (PI3K-Akt) and the Raf/Mek/Erk MAPK pathways mediate the very large spectrum of insulin actions. In insulin-sensitive tissues, insulin signaling via PI3K-Akt stimulates the membrane localization of insulin responsive glucose transporter GLUT4 (*SLC2A4* gene) to induce glucose uptake. Both PI3K-Akt and Raf/Mek/Erk intracellular cascades have numerous cytoplasmic and nuclear targets and impact a broad range of cellular processes. In the brain insulin, along with leptin, leads to increased expression of appetite-decreasing and reduced expression of appetite-stimulating genes. Impaired insulin signaling could have the opposite effect. Insulin, glucagon, other preproglucagon derived peptides, islet and/or gut hormones and the nervous system mediate the nutrient sensing, responses and energy expenditure outcomes [10], [11], [12], [13], [14], [15], [16], [17].

### 1.3 Glucose Uptake

Glucose uptake in the insulin responsive tissues is mediated by the product of *SLC2A4* gene, GLUT4. In the basal state, the transporter is sequestered in specialized vesicles that cycle/recycle between various compartments. Insulin signaling, described above, activates two downstream intracellular cascades, of which PI3K-Akt via AKT2—one of the three Akt members, modulates the activity of Rab proteins. The Rab family of small monomeric G-proteins regulates vesicular trafficking. Their function is modulated by the guanine exchange factors (GEF) that promote GDP to GTP exchange and the GTPase activating proteins (GAP) that promote GTP hydrolysis, to prompt or inhibit their activity, respectively. Potential GAPs, such as TBC1D4 (known as AS160), are substrates for AKT2; TBC1D4 phosphorylation by AKT2 inactivates it, thus terminating the inhibitory effect this GAP exerts upon Rab activity. Rho represents another family of small G proteins that regulate the cytoskeleton. Rac1 member, downstream of PI3K but independent of Akt, is involved in the trafficking of the transporter. Both arms of the transport pathway—the Akt-dependent and the Akt-independent, are necessary for proper trafficking. In adipocytes, insulin stimulated GLUT4 uptake involves another Rho member—Rhoq; it does not require PI3K but is dependent upon formation of a Rhoq activating complex downstream of insulin-stimulated insulin receptor [18], [19].

Reduced insulin-stimulated glucose transport is observed in T2D patients and animal models such as the obese Zucker rat. While in these cases, the impaired glucose transport is independent of GLUT4 expression, other models show reduced levels of GLUT4 in adipose tissue and skeletal muscle. The streptozotocin-induced diabetic rat, an experimental model of diabetes, shows insulin resistance and reduced Glut4 protein expression in skeletal and cardiac muscle, also seen in the Goto–Kakizaki rat model [20], [21]. Differential epigenetic inputs can result in varied levels, absence or presence of transcripts. Differential miRNA (microRNA) contributions can impact the level of protein products [4], [5]. Expression of *Glut4* in skeletal muscle is regulated by the activities of calcium/calmodulin dependent kinase 2 (CAMKII) and also by adenosine monophosphate-activated protein kinase (AMPK) and calcineurin-mediated signaling [22]. How a high-fat diet/obesity impacts on *Glut4* expression and/or translocation and activity and, in a broader sense, on the lack of adequate insulin response in the presence of high glucose, at the molecular level, remains to be resolved. Obesity is a known risk factor in the development of T2D. Overexpression of *Glut4* in the adipose and skeletal muscle tissues of transgenic mice improves insulin-stimulated glucose uptake and diminishes the insulin resistance exhibited in T2D and obese states [21].

### 1.4 The Versatile Glucose

Glucose, the six-carbon sugar, may not be as potent an energy source as the larger fatty acids molecules, but is a major and the primary fuel of the brain and a versatile molecule overall. A small percentage of glucose is used in the hexosamine biosynthetic pathway. Its major product, uridine diphosphate N-acetylglucosamine (UDP-GlcNAc) is used for the O-GlcNAcylation of many cytoplasmic and nuclear proteins. The serine and threonine glycosylation sites can also be phosphorylation sites by serine/threonine kinases. The high incidence of O-GlcNAcylation and its interplay with phosphorylation modulates many physiological processes and plays a role in the etiology of diseases that includes diabetes, cardiovascular and neurodegenerative conditions and cancer. Hexosamine biosynthesis engages glucose, amino acid, fatty acid, and nucleotide metabolism and as such is considered a nutrient sensor. Its role in pancreas development and beta-cell physiology coupled with the high levels of enzymes and modified proteins underlies the potential role it has in diabetes. Increased levels of O-GlcNac levels appear to be involved in insulin resistance and diabetes development [23], [24].

An alternate route of glucose oxidation is the pentose phosphate pathway (PPP), also known as the pentose phosphate shunt. The initial oxidative branch produces NADPH necessary for antioxidant and anabolic pathways and other essential cellular systems. The non-oxidative branch produces ribose 5-phosphate, required for nucleic acid synthesis. Given the range of cellular demands for NADPH, the importance of glucose-6-phosphate dehydrogenase (G6PD), the first and rate-limiting enzyme in the PPP, is not surprising. Studies show decreased G6PD activity, in many tissues, resulting from hyperglycemic or diabetic states. Decreased G6PD activity in the kidney cortical cells of streptozotocin-induced diabetic rats and decreased beta cell survival in mouse and human islets have been reported [25].

The main product of glucose oxidation—pyruvate—is at the cross roads of pathways important for glucose and energy homeostasis. Its irreversible decarboxylation to acetyl-CoA can fuel the tricarboxylic acid cycle or be used for fatty acid biosynthesis. Pyruvate shuttling to and from the mitochondrion is mediated by three main cycling pathways via oxaloacetate (OAA) conversions. Pyruvate cycling may play a role in the second phase of insulin secretion. The pyruvate cycle is perturbed in models of T2D and dysfunctional islets, such as the prediabetic Zucker diabetic fatty (ZDF) rats [26].

Given the range of hormonal actions and the critical role of glucose homeostasis and its relationship to energy and cellular homeostasis, the complexity of the diabetic condition and the difficulty of deciphering its routes at the molecular level are of no surprise. In the following sections pertinent information available at RGD is investigated, existing or newer directions are presented, possible ramifications are discussed.

## 2 Materials and Methods

RGD runs on an Oracle database. The tools built by RGD use J2EE technologies and they can be run in any Java container implementing Java Servlets and Java Server Pages. Application web development relies on Spring's model-view-controller (MVC). User interface relies on Ajax and Javascript and Cascading Style Sheets (CSS). Pathway diagrams are built with Pathway Studio software package from Elsevier, version 10. Pathway diagram pages are built via a web application specifically developed at RGD [27, see also 29, 31, further down].

## 3 Results and Discussion

### 3.1 Starting the Journey

#### 3.1.1 Entry Points—Disease and Pathway Portals

**Fig. 1:**
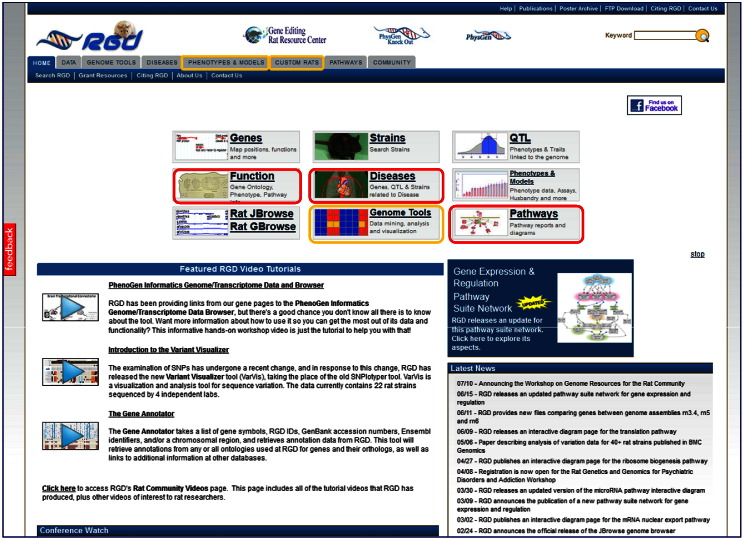
RGD home page. The main home page of RGD with the various entry points. The Disease and Pathway Portals, and the Function options are highlighted in red; the Genome Tools and the Phenotypes and Models portal, and the Custom Rats entry, are highlighted in yellow. Diseases and Pathways options, and the Genome Tools, are accessible from both the icons and the tabs at the top of the page.

**Fig. 2:**
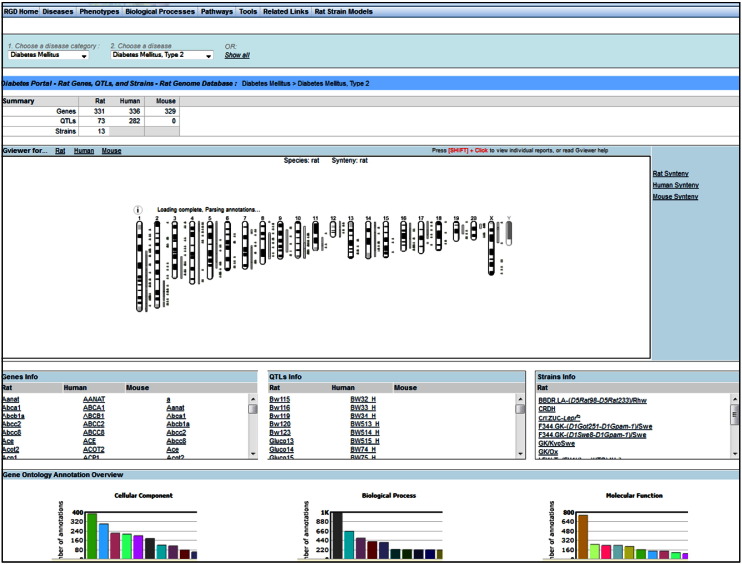
Example of a disease portal query. A result using the Diabetes Disease Portal, specifically looking for type 2 diabetes mellitus in the diabetes mellitus category, showing the summary of annotated objects on the top, followed by GViewer, the itemized lists of annotated objects and a partial view of Gene Ontology overview.

There are several entry points on the main homepage of RGD to access data for topics of interest, here diabetes. Disease and Pathway Portals look like good choices to start the journey **[**Fig. 1, **highlighted in red]**. “Diseases”, currently home to nine portals, includes cancer, cardiovascular, diabetes, immune and inflammatory, neurological, obesity/metabolic syndrome, renal, respiratory and sensory organ [28]. For each portal, the disease data option is the default. The other options include phenotypes, biological processes, pathways, tools, related links and rat strain models. For any option one can select vocabulary/ontology terms from a broad category and then select any of the more specific terms within the broader category. For each selection, a summary table shows the total of annotated objects—genes, QTLs and strains, by species, as applicable. Itemized lists of genes and QTLs for rat, human and mouse, and for rat strains are displayed. Every entry links to its corresponding report page in RGD. A Genome Viewer (GViewer) offering a genome-wide view of annotated rat genes, QTLs and strains, and an overview of Gene Ontology (GO) annotations across the three vocabularies of GO are also present. In Fig. 2, a selection is made for the category ‘Diabetes Mellitus’ and for the disease term ‘type 2 diabetes mellitus’, and the results are shown. As mentioned, there is a range of complications associated with diabetes affecting various systems. They can be found in the diabetes portal, under the category ‘Diabetes Complications’ as well as in the portals dedicated to those systems.

**Fig. 3:**
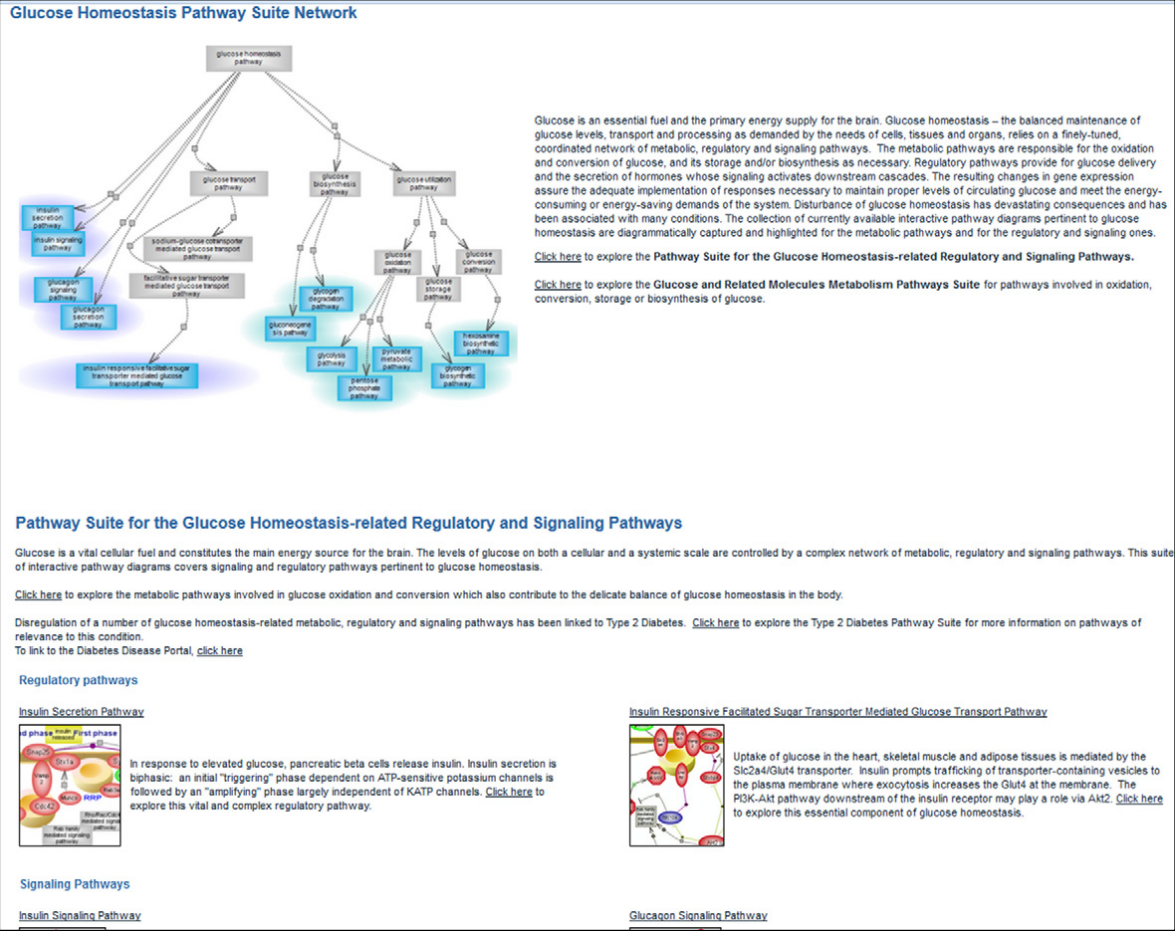
Glucose homeostasis pathway suite network. The glucose homeostasis pathway suite network, partial view. A graphical overview and short description are on top of the page. From within each pathway suite, the individual diagram page(s) can be accessed by clicking on their image(s), the title or the underlined ‘click here’ option.

The Pathway Portal offers a collection of interactive diagram pages organized by the five major nodes of the pathway ontology (PW): classic metabolic, signaling, regulatory, disease and drug pathways. In addition, there are terms and associated diagrams for the altered version(s) of pathway(s). A disease pathway is viewed as the intersection of altered pathways converging into the disease phenotype [29], [30]. Many, if not most, of the pathways of glucose metabolism have interactive diagram pages while others are dedicated to insulin secretion, insulin and glucagon signaling and the downstream cascades they activate, glucose uptake, altered pathways relevant to T2D, the T2D disease pathway and the pharmacokinetics and pharmacodynamics pathways of several drugs used in T2D treatment. The portal also offers pathway suites and suite networks. A pathway suite or suite network offers an instant snapshot of pathways connected within a broader context or of suites that are inter-related, respectively. For instance, the two pathway suites within the glucose homeostasis pathway suite network are for the glucose-related regulatory and signaling pathways and for the metabolism of glucose and related molecules, respectively. They are differentially highlighted in the overall representation. Each entry within the two suites links to that individual diagram page **[**Fig. 3**]**.

#### 3.1.2 Tools

**Fig. 4:**
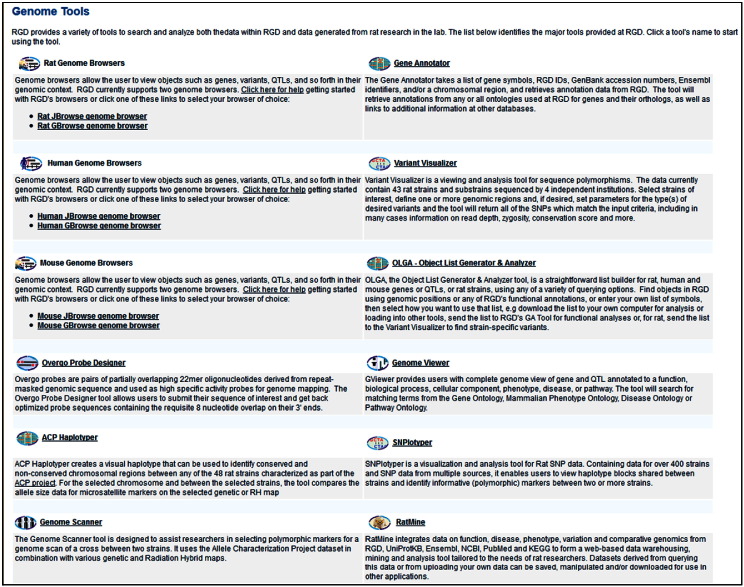
Genome Tools. The list page of currently available tools, each with a brief description and link to the individual tool page. The page can be accessed from ‘Genome Tools’ entry on the main homepage.

RGD offers a collection of tools, most built-in-house, to allow for the retrieval, visualization and analyses of data [31]. The ‘Genome Tool’ entry point **[**Fig. 1, **highlighted in yellow]** presents a succinct description of them with links to the sites [Fig. 4]; some will be used as entry points, others during the more involved, exploratory leg of the journey. The ‘Phenotypes & Models’ portal provides rat phenotype and rat strains and disease models data and also contains the PhenoMiner database which houses quantitative records of phenotype data **[see**Fig. 1, **highlighted in yellow]**. Video tutorials are available for help with how to use several of these tools. The growing collection of videos is available from the main homepage of RGD. A recently added video tutorial is for the “Object List Generator & Analyzer” OLGA tool; the tool will be used during the exploratory step of this journey.

##### 3.1.2.1 Genome Tools—GViewer

**Fig. 5:**
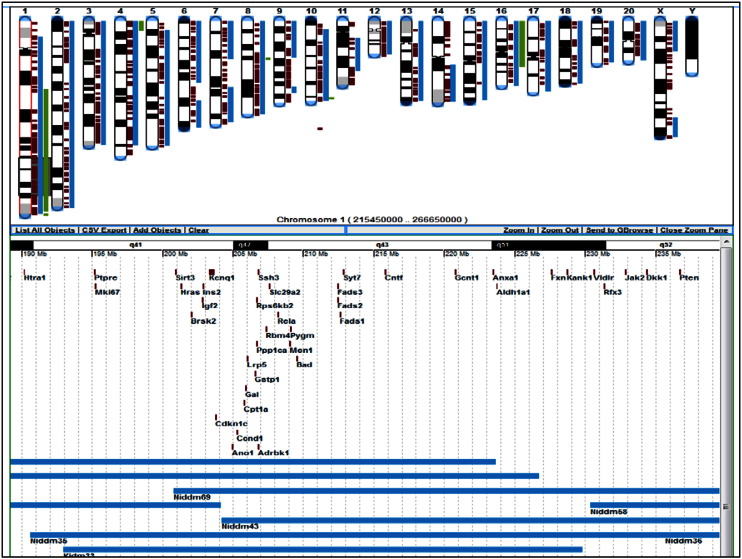
Genome Viewer (GViewer). A GViewer result for ‘insulin’ keyword search, showing the zoom pane for ‘gliding’ along chromosome 1.

The ‘Function’ entry point **[see**Fig. 1, **highlighted in red]** and GViewer in Genome Tools **[see**Fig. 4**]**, both allow for ontology searches to retrieve results for terms of possible interest and their associated annotations using user-selected keywords. A ‘Function’ result will show a summary of the number of terms that match a given keyword in each ontology and whether there are annotations. Once a selection is made, that particular ontology can be searched in the ontology browser. In GViewer one can search all (default) or a subset of ontologies. The result will return the objects and the term(s) they are annotated to containing the keyword(s) used in the query, along with a genome-wide view (GViewer) of rat genes, QTLs and strains annotated to those terms and their chromosomal association. For instance, typing ‘insulin’ in the search box with the default option of all, brings up over 1100 genes, some 250 QTL and 9 strains (genes are colored brown, QTLs are in blue, strains in green). One can ‘glide’ along individual chromosomes by clicking on one, and move along its length. A zoom pane will open showing the objects in the areas ‘scanned’ horizontally, where one can zoom in and out, access the individual report pages for the objects within by clicking on them, go to the Genome Browser (GBrowse) for more options, or close the pane. **[**Fig. 5**]**. Report pages can also be accessed from the tabular list of objects located below the GViewer (and the pane, if opened) or by right clicking on the object in the GViewer. In addition to viewing the report page, the right click also gives the options to access the object in GBrowse or to remove it from the GViewer display. The tool also offers the option of adding search terms using Boolean operators (default is OR). Selecting ‘glucose’ for instance, with the AND operator keeps the list still fairly large, as expected. One could do the same search(es) but select for ontologies related to clinical phenotype, such as the clinical measurement ontology (CMO), the measurement method ontology (MMO) or the experimental condition ontology (XCO). However, in these cases, it would be interesting to view the quantitative phenotype data, using the PhenoMiner.

##### 3.1.2.2 PhenoMiner

**Fig. 6:**
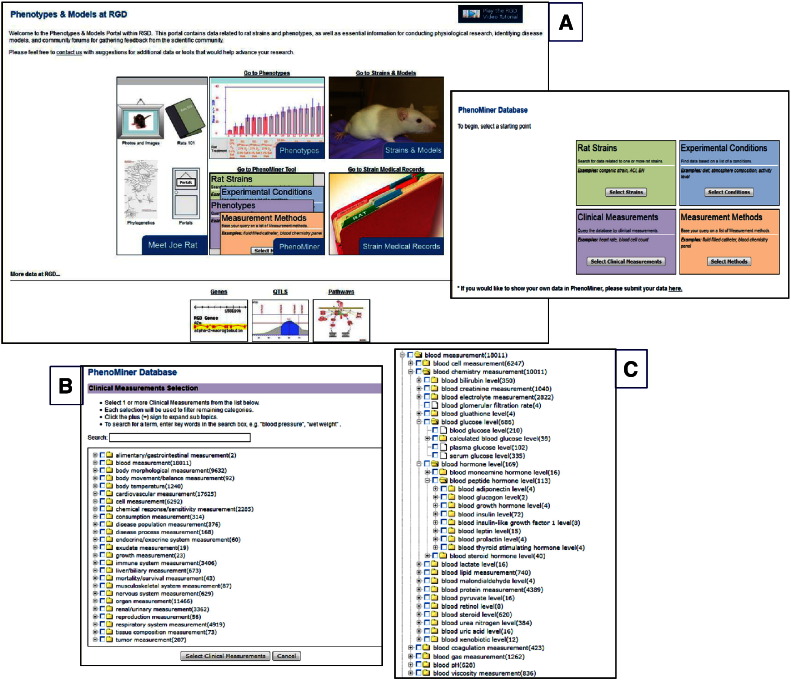
Phenotypes & Models, PhenoMiner. An overview of Phenotypes and Models showing the entry point to PhenoMiner database (A and inset), the main nodes in CMO (B) and expansion of selected nodes to view more specific terms (C).

**Fig. 7:**
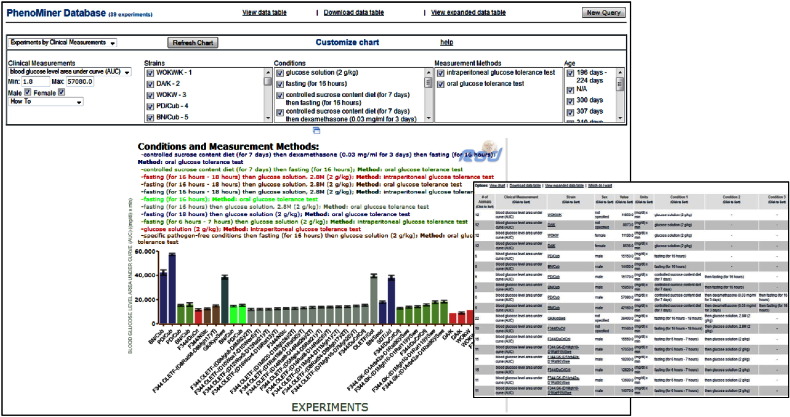
PhenoMiner report. The PhenoMiner report for ‘calculated blood glucose level’ term and a view of the expanded table in the inset.

The PhenoMiner database, at ‘Phenotypes & Models’ **[**Fig. 1, **highlighted in yellow]**, is specifically designed to integrate rat phenotype measurements and phenotypic data from multiple studies—http://rgd.mcw.edu/phenotypes/[32], [33]. PhenoMiner can be queried using one or a combination of several ontologies/controlled vocabularies, developed at RGD, some specifically designed for the PhenoMiner project [34]. The four ontologies/vocabularies for querying the database are: CMO, MMO, XCO and Rat Strain (RS) **[**Fig. 6A**and inset]**. CMO—the clinical measurement ontology is developed to incorporate terms for what is actually being measured, e.g., circulating metabolites, organ/tissue measurements, including diseased organ/tissue measurements; MMO—the measurement method ontology is developed to incorporate terms for the method used to make the measurement, e.g., radiotelemetry, radioimmunoassay; XCO—the experimental condition ontology is being developed to incorporate terms pertinent to the condition(s) of the experiment for which the measurement is done, e.g., elements in the diet, in the environment. In the example, CMO is used to find terms associated with insulin or glucose levels or for terms pertinent to pancreatic islet cell measurement by selecting ‘blood measurement’ or ‘organ measurement’ nodes and navigating further down. For instance, by expanding the ‘blood chemistry measurement’ child term, one can see the various terms for glucose levels or those under blood hormone/blood peptide level for insulin or other hormones **[**Fig. 6B and C**]**. The number of records for each term is shown in parentheses to the right of terms. The results can be further narrowed by selecting terms from the other vocabularies or they can be viewed by requesting the report. Results are shown graphically and also within a table, which can be downloaded or viewed in expanded mode for additional details **[**Fig. 7**and inset]**. From the expanded view one also has the option to access the references for results of interest, such as those associated with glucose levels in the Goto–Kakizaki (GK) derived strains. GK is an extensively used model for T2D [35]. Congenic strains carrying segments of GK chromosome 1 in the F344 normoglycemic background are established; QTLs define regions on chromosome 1 associated with defective insulin secretion and with signs of insulin resistance. The impaired insulin secretion locus on rat chromosome 1 is conserved across species; syntenic regions harbor T2D candidate genes such as the human *TCF7L2* or mouse *Sorcs1.* Niddm35 (non-insulin dependent diabetes mellitus rat QTL 35) and the short Niddm36 (non-insulin dependent diabetes mellitus rat QTL 36) covering a contiguous region of chromosome 1 and overlapped by Niddm43 (non-insulin dependent diabetes mellitus rat QTL43), are amongst the loci in the region [36], [37], [38], [39]. At this point, perhaps one should embark on an exploratory journey, using the QTLs and the candidate genes as starting points, to navigate resources and tools at RGD, accompanied by the available literature and bearing in mind the various aspects of glucose homeostasis along with the roles of epigenetics and of non-coding elements, as they likely combine in the complex etiology of T2D and of diabetes in general.

### 3.2 An Exploratory Journey

#### 3.2.1 From QTL to Genes, Using the OLGA Tool

**Fig. 8:**
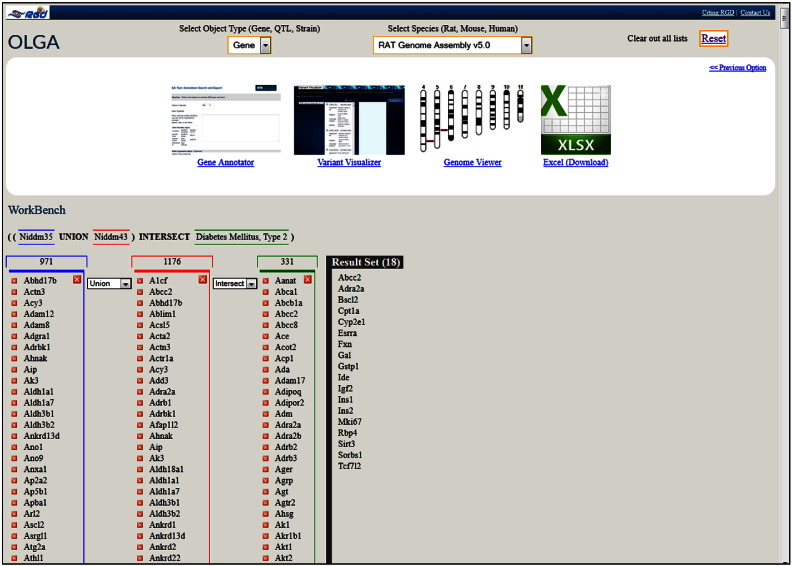
Object List Generator & Analyzer (OLGA). OLGA result list for the union of QTLs Niddm35 and Niddm43 intersected with Type 2 Diabetes Mellitus and the options available for its further analysis or download.

The three diabetes-associated rat QTLs identified above using the PhenoMiner tool: Niddm35 and Niddm36 occupy adjacent regions of rat chromosome 1 whose span is overlapped by Niddm43, which also overlaps many other QTLs. Of these, a substantial number are associated with glucose and lipid levels, body weight and renal function followed by those associated with blood pressure, cardiac and bone features. Also present, are several tumor susceptibility QTLs. A great number of genes are in the regions spanned by these QTLs. While these types of data can be accessed by selecting the ‘QTLs in Region’ and ‘Genes in Region’ options available in the QTL report pages, the ‘Object List Generator & Analyzer’ (OLGA) tool **[see**Fig. 4**]** offers a variety of querying and data manipulation options. Lists of rat, human and mouse genes or of QTLs and rat strains can be generated using one of several options; for instance, ontology term or genomic region. The lists can then be used/combined with intersection, union or subtraction options; the resulting list can be downloaded or sent to other tools for further analysis. In Fig. 8, the QTLs Niddm35 and Niddm43 were chosen and the generated gene lists were used in conjunction with the union option. The new list, containing 1,428 unique genes, was then intersected with the list of genes generated for the T2D term in the disease ontology, containing 331 genes. The resulting set contains 18 genes and several options are available for its further use; the set can be downloaded for later analysis or sent directly to other tools, such as the Gene Annotator (GA) or the Variant Visualizer**[**Fig. 8, **upper frame]**. In the GA tool one can look up the available annotations for any or all the ontologies as well as information available in other databases. Variant Visualizer lets one choose one or all sequenced rat strains [40], [41], and select parameters (default is all) to return variant data. Both tools will be used as the exploration continues.

#### 3.2.2 A Set of Genes

A quick inspection of the function/process and pathway involvement of the eighteen genes using the GA tool, shows a spectrum of biological roles. It has to be mentioned here that ontology-based results incur a bias if genes happen not to be annotated to queried terms—genes may not be annotated because there is no available data in the literature, because data is suggestive but not conclusive enough, or because in the curation process, the genes have not yet been targeted (RGD targets genes based on their disease association for portal specific conditions and relevant pathways). Of the two genes mentioned earlier—*TCF7L2* and *Sorcs1*, only rat *Tcf7l2* is on OLGA's result list, as only *Tcf7l2* has a T2D annotation. It is likely that for *Sorcs1* there is little data in the literature; inspection of Gene Ontology (GO) annotations shows that there is a paucity of them for this gene across the three species. *Sorcs1* is not annotated to T2D but it is a candidate gene for the QTL Niddm65 (non-insulin dependent diabetes mellitus QTL 65), which is annotated to T2D and resides within the species-conserved region of GK strain chromosome 1 associated with T2D susceptibility. The mouse *Sorcs1* gene is spanned by several QTL, including the ‘type 2 diabetes mellitus 2’, T2dm2_m. Note that T2dm2_m (and other diabetes related mouse QTLs) has the disease in its name. Mouse phenotype annotations, using the mammalian phenotype (MP) ontology, for both the gene and the QTL were made by the Mouse Genome Informatics (MGI) database and imported via the mouse MP pipeline.

Of the 18 genes, several have a role in drug transport and metabolism (*Abcc2*, *Cyp2e1* and *Gstp1*), two are the insulin genes (there are two in rodents) or the insulin-like growth factor (*Igf2*), others are involved in lipid and vitamin metabolism (*Cpt1a*, *Bscl2* and *Rbp4*), histone modification (*Sirt3*) or, like *Tcf7l2*, play a role in transcription (*Esrra*). Interestingly, some have a role in the nervous system such as *Gal*—the ligand for galanin receptor, involved in action potential, the adrenergic receptor *Adra2a*, or *Ide*—the insulin degrading enzyme, which also degrades glucagon and the amyloid beta peptide underlying the pathogenicity of Alzheimer Disease. Most genes are implicated in a range of conditions that collectively, in addition to T2D, include obesity, cardiac and vision disease, heart and kidney failure, neurodegenerative disease and various cancers. Perhaps, the time is ‘ripe’ to investigate the *TCF7L2* gene story in more detail.

#### 3.2.3 The *TCF7L2* Gene

TCF7L2 is a transcription factor and a major effector of the beta-catenin dependent pathway of Wnt signaling, known as the canonical pathway. In the absence of a Wnt signal, beta-catenin is sequestered into the cytoplasm by a ‘destruction complex’ phosphorylated and targeted to degradation. Wnt signaling promotes destabilization of the destruction complex; beta-catenin can then translocate to the nucleus to displace repressors from the Tcf/Lef effector complex and recruit a number of co-activators. Alterations in components of the pathway have been implicated in several conditions, primarily tumorigenesis and bone malformation. The range of Wnt target genes is rather large which is not surprising given the many roles the pathway plays in embryonic development, tissue regeneration, cell polarity, proliferation and cell fate determination [42—the interactive diagram page for Wnt signaling, canonical pathway, has tabular views of its genes and their involvement in diseases and pathways, direct link to the diagram page for its altered version, references and graph path view]. Studies in rodent and human pancreatic islets, show that TCF7L2 directly and indirectly impacts on a transcriptional network important for the proper maintenance of beta cell and the production of insulin. Primarily via ISL1 (islet 1) transcription factor, it regulates downstream effectors that include transcription factors, proinsulin processing enzymes PCSK1 and PCSK2, and SLC30A8 (ZnT8) zinc transporter. The transcription factors, which include MAFA, PDX1, NKX6-1 and also NEUROD1, are known for their roles in the maintenance of beta cells and insulin biosynthesis [43], [44]. SLC30A8 (ZnT8) is almost exclusively, and also abundantly, expressed in the islets of Langerhans, and is associated with insulin secretory granules. Zinc is essential for the formation of proinsulin hexamers which in the immature secretory granules will be processed into zinc–insulin hexamers, characteristics of the mature granule. Polymorphisms in the transporter gene differentially influence T2D with both increased and decreased disease risk [45], [46].

**Fig. 9:**
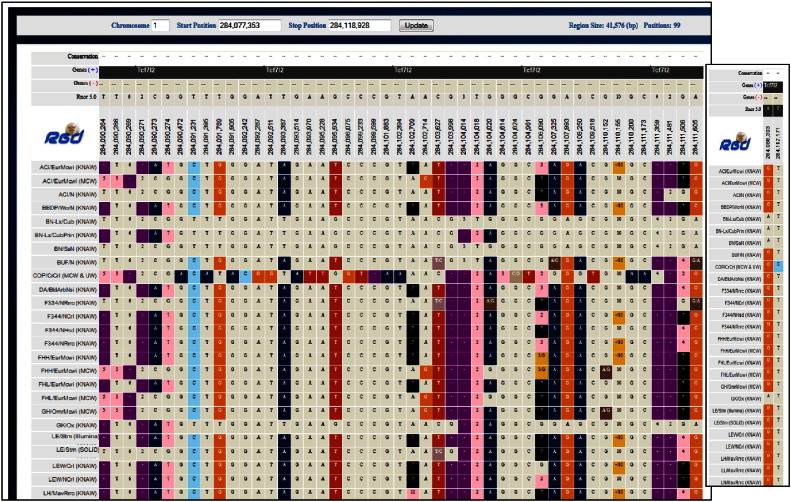
Variant Visualizer. Results for *Tcf7l2* in Variant Visualizer with all strains selected and the default parameter options (all); the inset shows the two exonic variants in the GK strain.

The gene report page for the human *TCF7L2* gene shows disease annotations to T2D as well as to gestational diabetes, diabetic nephropathies, insulin resistance and pancreatic neoplasms disease terms and to pancreatic cancer pathway term. In addition, there are several other cancer pathway annotations based on annotations from KEGG pipeline [29]. T2D annotations are also imported from NCBI's ClinVar and OMIM via dedicated pipelines. Looking up the human variants from ClinVar Clinical Variants section under Sequence category on the gene report page, one can see that there are several copy number loss or gain variants, and some single nucleotide polymorphism (SNP) variants. Most are categorized as pathogenic or likely pathogenic, for phenotypes unrelated to T2D, few (the SNPs) are categorized as T2D risk factors. Choosing the coordinates of rat *Tcf7l2* and all sequenced strains in Variant Visualizer with the default parameter options (all) shows that, except for two variants which are exonic (and synonymous), all variants are outside exons and there are many insertion/deletions, as evidenced by the presence of numbers/minus sign within the colored boxes (a colored box indicates a difference from the reference position) **[**Fig. 9**and inset]**. Insertion/deletions can change the reading frame and non-exonic variants can affect splicing. There are several transcripts of the human gene, with alternative splicing producing a number of isoforms. Alternative splicing of the human *TCF7L2* gene exhibits both general and tissue-specific features, the latter including the pancreatic islets where transcripts containing exon4 are at twice the level observed in other tissues. The alternative exon4 is one of several alternative exons in *TCF7L2*; interestingly, the T2D risk allele is intronic [47].

#### 3.2.4 Downstream of *TCF7L2*

As mentioned, in the pancreatic islets TCF7L2 directly regulates ISL1, a member of the LIM/homeodomain family of transcription factors, and impacts further downstream the MAFA, PDX1, NKX6-1 and NEUROD1 transcription factors, the proinsulin processing enzymes PCSK1 and PCSK2 and the zinc transporter SLC30A8. The transcription factors play essential roles in the formation and maintenance of mature beta cell function and the expression of the insulin gene. Chronic exposure to glucose, possibly exerting an oxidative stress effect, affects the expression and/or DNA binding activity of PDX1 and MAFA, an effect alleviated by antioxidant treatment [44]. Checking the disease annotations for *Pdx1* and *Neurod1* genes in the database, one finds manual annotations to T2D, as well as glucose intolerance and insulin resistance, hyperglycemia and obesity*. Nkx6-1* has one disease annotation to spinal cord injuries, and the human gene is a candidate gene for GLUCO232 (glucose level QTL 232). Mafa genes do not have disease annotations. All four transcription factors have KEGG annotations to the ‘maturity onset diabetes of the young pathway’ term, while *Pdx1* and *Mafa* are also annotated to the T2D pathway. Interestingly, NEUROD1 and possibly PDX1 may have a role in the transcription of MIR-375—a microRNA associated with pancreatic function [48]. PDX1 may exert upstream regulatory roles whereas NEUROD1 interacts with both upstream and downstream conserved sequences. The promoter region of *MIR-375* contains binding sites for ONECUT1, (also known as HNF6) and INSM1 transcription factors, both important for the development of pancreatic islets. Of the two genes, *Onecut1* is associated with the maturity onset diabetes of the young pathway (KEGG pipeline) and both appear to be involved in pancreatic islet function.

What is the role of Mir-375 and of non-coding RNA molecules in general, in the context of pancreatic islet function or dysfunction, insulin and associated glucose homeostasis?

#### 3.2.5 Non-Coding RNA and the Islet Cells

It is now known that the genome is pervasively transcribed and that non-coding RNAs exert important regulatory roles. The microRNAs (miRNAs) generally inhibit the translation of target genes, while the long non-coding RNAs (lncRNAs) span a range of regulatory functions [49], [50], [51].

##### 3.2.5.1 miRNAs

Mir375, specifically expressed in islet cells, is believed to play a role in the early stages of islet development, particularly as the embryonic stem cells differentiate into liver and insulin secretory cells [48]. Mir375 is also involved in regulating insulin expression and secretion. And Mir375 is one of a number of miRNAs involved in insulin synthesis and secretion (for instance Mir9 and Mir29a/b/c), insulin sensitivity in target tissue (Mir143 and Mir29) or glucose and lipid metabolism (Mir103/107 and Mir122) and thus, having potential roles in diabetes [see for instance, [52], [53]. Several, including Mir375, are differentially expressed in T2D patients and rodent models such as the obese diabetic mouse and the GK rat.

miRNAs can have a large number of mRNA targets and a single mRNA may be the target of many miRNAs; the number of predicted targets is indeed very large. However, experiments continue to increase the number of validated targets, thus helping to delineate the regulatory realm of individual miRNAs. Validated and predicted mRNA targets for human, mouse and rat miRNAs can be found at the miRGate database [54], and now in RGD.

**Fig. 10:**
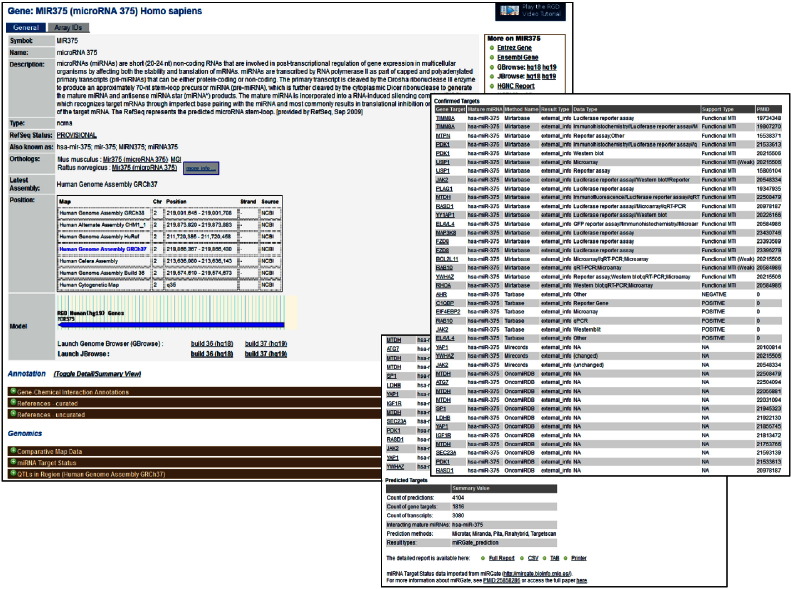
A miRNA gene report page. The report page for the human MIR375 gene; the insets show the validated targets and the overall target information.

A newly added feature at RGD, is the miRGate data import. Users can search for the miRNA of interest across the three species, then scroll down the gene report page to the Genomics section where the miRNA Target Status option is located. Clicking on the option displays the results—if there are validated targets, those are displayed; a tabular report for all targets is also available and the detailed report can be downloaded in several formats. The results for the human MIR375 are shown **[**Fig. 10**and insets]**. The only validated mRNA target of rat Mir375 is Pdk1, also validated for the human and mouse microRNA 375. In addition, the larger list of mRNA targets of mouse miR-375 is comparable to the human list, in terms of gene. This is reassuring as it points to a comparable, and conserved, miRNA regulatory network, despite the differences between the cell composition and the spatial architecture of human and rodent—mouse and rat islets are the two extensively used models of pancreatic function and functional failure. As a note, the human islets comprise ~ 50% beta, ~ 40% alpha and ~ 10% delta cells, the remainder being epsilon and PP cells, with a random-like distribution. In contrast, rodent islets are 60–80% beta, found in the core and surrounded by 15–20% alpha cells in a mantle fashion [55].

The reciprocal miRNA information for the genes, which miRNA targets them, is similarly available on the gene report page. Only mouse *Tcf7l2* has a validated miRNA for which it is the target—Mir210, which has only a human ortholog currently displayed in RGD.

##### 3.2.5.2 lncRNAs

Long non-coding RNAs, by definition larger than 200 nucleotides but many times spanning very large regions have been assigned a spectrum of regulatory roles, from transcriptional regulation with both activating and repressive inputs, to scaffolding and architectural determinants, chromatin dynamics, epigenetics, higher-order chromosomal organization [56]. An inventory of the human beta cell transcriptome indicates that islet lncRNA are tissue-specific and also stage-specific. While some locate in genomic regions devoid of protein-coding genes, others map to regions of protein-coding genes important for beta cell function, many in divergent, *antisense*, orientation [57]. Interestingly, some are differentially expressed if cells have been exposed to increasing glucose concentration (*HI-LNC78*, and *80*, official nomenclature *TUNAR* and *OLMALINC*, respectively); others are aberrantly expressed in the islets of T2D donors. As expected, glucose exposure also increases the expression of genes involved in insulin dependent function/responses, including insulin (INS) itself.

Inspection of RGD entries for the two human lncRNAs cited above, reveals that there is little data available. Perhaps not surprisingly, inspection of QTLs in the regions reveal that they are associated with glucose levels and body weight, and in the case of TUNAR, also with insulin and leptin levels. Interestingly, there are also tumor related QTLs in these regions: prostate tumor susceptibility for both lncRNAs, also mammary tumor susceptibility for TUNAR. Of the two, only TUNAR has an ortholog in the mouse system. However, sequence similarity may not be a particularly stringent requirement for lncRNA function if the secondary/tertiary structural features are preserved. Unlike the protein coding genes, dependent on the three-letter amino acid specification of codons for conserved peptide composition, dictating the structure and subsequent function of the protein, the folding of non-coding RNA is dependent on whether base-pairing is thermodynamically favorable, rather than the identity of the nucleotide.

The high tissue specificity of coding and non-coding islet genes has to be tightly linked to the chromatin organization of those loci, and thus to the epigenetics that control them. Indeed, the CpG islands of *INS* in non-insulin producing cells (most others) are methylated and therefore do not express insulin; they are demethylated in the insulin-producing pancreatic islet beta cells [5 and references within]. With the genetic components of T2D being so elusive, perhaps some of the contributions to and/or susceptibilities for the condition rise from an unbalanced or deregulated epigenetic transcriptional control. Is there evidence for it, and if so, what might be the mechanisms?

### 3.3 Epigenetics and Diabetes

Epigenetic changes which involve the modification and remodeling of chromatin, are heritable and independent of DNA sequence, and they play a key role in the differential expression of genes. The methylation of DNA at cysteine residues (CpG) and the many ways in which the histone tails are modified represent the modification route; the remodeling route is dependent on four protein complexes representing four distinct pathways which can space, slide or reposition entire nucleosomes as well as insert or evict histones, in an ATP-dependent fashion. All, are components of one of the suites within the ‘Gene Expression and Regulation Pathway Suite Network’ [58] in the ‘RGD Pathway Suites and Suite Networks’ collection; this suite network has been recently updated.

The differential methylation of the insulin gene has been mentioned [5]. As the promoter of INS is highly methylated in all other cells except the islet beta cells, the death of beta cells will lead to its components, such as unmethylated *INS*, to be present in the circulatory system. Detection of unmethylated INS in the circulation could be a means to assess beta cell death resulting from normal turnover of the cells or possibly, as is in the case of T1D, aberrant cell death. This approach has been used in mouse models of T1D. Aberrant methylation in islets from T2D donors versus healthy individuals, as well as in skeletal muscle and adipose tissue is observed, although it is not clear whether this is the result or the cause of the condition. Single nucleotide polymorphisms (SNPs) from T2D donors that either remove or introduce a CpG site, are also reported [59] Many studies have addressed the issue of DNA methylation and changes from its regular patterns in diabetes, but histone modification and its aberrant phenotype in diseases, including diabetes, is also reported. Metabolism/diet can affect epigenetics via provision of donor groups; for instance, the methyl group via the methionine cycle, the UDP-GlcNAc via the hexosamine pathway or the acetyl group from acetyl-CoA via either pyruvate metabolism or fatty acid degradation. Obesity, highly prevalent in parts of the world and increasing throughout, is associated with diabetes and cardiovascular conditions. Diet-induced obesity can alter chromatin modification in rodents and as mentioned above, there are epigenetic alterations in T2D donors [60, also 5, 24]. Differential gene expression and associated changes in chromatin modification are observed in obese prone (OP) versus obese resistant (OR) rats fed a high-fat (HF) diet [61]. But epigenetics and epigenome–environment connections are also implicated in the host–microbiota relationship and data increasingly suggest that this is a two-way road. Developmental programs, such as those of immune cells, are shaped through differential genes expression; the immune system is key to shaping the microbiota; microbiota-derived molecules impact on the epigenome of the host and the resulting functional effects reflect back on the environment that modulates the microbiota [62]. What is the role of microbiota in diabetes?

### 3.4 Microbiota and Diabetes

The cross-talk between the immune system and microbiota and the connection between autoimmunity and T1D suggests that the microbiota likely has a role in the condition. Studies show that there are differences in gut microbiota, composition and/or function between diagnosed and healthy children, although it is not clear if this underlies the etiology of the disease or reflects the host-commensal microbe selections [see 6]. Although T1D has long been associated with adaptive immunity, studies reveal that there is an innate inflammatory response in the biobreeding (BB) rat strain models of T1D and humans with T1D. It is thought that gut microbiota may have a role in shaping adaptive immunity and could extend beyond the gut [see 6]. Changes in gut microbiota can result in intestinal permeability and proinflammatory responses, as reported for human and animal models [63].

While the link between T2D and the microbiome may be rather elusive, its connection to obesity is well evidenced. The composition of gut microbiota is different between obese-prone (OP) and obese-resistant (OR) rats, BB diabetes-prone and diabetes resistant (BB DP versus BB DR) rats and also, between obese mice or human subjects, and the lean individuals [see 6, 62]. It has to be mentioned that there is diversity between the microbiome of different individuals and even greater diversity between the sites the microorganisms occupy [64].

## 4 Conclusions and Future Directions

### 4.1 Continuing the Journey

In the previous sections, diabetes-related data, particularly T2D, were sought starting from selected entry points, navigating between and from strains, QTLs and genes and further expanding the investigating range using a few leads and, throughout the journey, employing several of the available tools. It was one course among others that could have been followed using the same ‘starting material’ but traveling along different routes.

Wnt signaling, for which TCF7L2 is one of the mediators, besides the genes downstream of TCF7L2 identified in pancreatic islets, has many other targets in many tissues, including components of the Wnt pathway itself (see the links from the target gene page, in the diagram, ref [42]). TCF7L2 also controls the expression of preproglucagon (*GCG*) in the brain and gut [65]. The processing of GCG gives rise to glucagon and several other peptides like somatostatin, glucagon-like peptide-1 and -2, all with roles in insulin and glucagon secretion, glucose and energy homeostasis. Which peptide is expressed where, depends on the relative levels of processing enzymes PCSK1 and 2, which in the islets, as mentioned, are under TCF7L2 control (and possibly, others). Could an imbalance in *GCG* outcomes, whose putative origins may range from sequence variation to non-coding RNA control, epigenetics, diet/environment interactions, and microbiota, in single and any combination of them, result in aberrant contributions to glucose levels and hyperglycemia? The contribution of glucagon to diabetes, that is glucagon excess, rather than insulin deficiency, has been proposed [16].

It should be mentioned here that rat *Tcf7l2*, and the exclusively pancreatic islet expressed *Slc30a8*, whose variants confer both diabetes risk and protective effects [46] and whose autoantibodies are detected in T1D patients [66], have targeted mutations introduced via zinc finger nuclease (ZFN) mutagenesis. These are two among many other genes targeted for knock-out in the PhysGen Knockouts program. The status of genes and strains, contact and other valuable information, can be found at the site, available as link from the strain report page or the ‘Custom Rats’ tab on the main home page **[**Fig. 1, **highlighted in yellow]**.

Interestingly, besides genes particularly if not uniquely expressed in beta cells, others appears to be ‘forbidden’, although rather abundantly expressed in other tissue types [13 and references therein]. Also, the pancreatic beta cells can dedifferentiate to a progenitor-like state and are able to convert to other hormone-producing cells. Results from animal model studies point to the role of *Foxo1* in the process, whose gradual loss in the context of progressing T2D can be pathogenic [67]. The gene is a member of the large forkhead box (FOX) family of transcription factors involved in a range of processes, including differentiation. The manual disease annotations for *Foxo1* point to both T1D and T2D associations, as well as insulin resistance, heart failure, premature aging and, like TCF7L2, to pancreatic neoplasms. The pathway annotations via the KEGG pipeline indicate insulin signaling and prostate cancer pathways while the manual annotations point to transforming growth factor beta (TGF-beta) SMAD dependent signaling, for which this and other FOX members are interacting co-factors/co-activators of SMADs. Like Wnt, TGF-beta SMAD dependent signaling is involved in embryonic development, cell proliferation, differentiation, among other processes. And while *Foxo1* is not one of the culprits in the altered version of the pathway, the altered TGF-beta SMAD network is associated with pancreatic cancer and also, with colorectal cancer pathways. Looking up the references for the metformin drug pathway, a T2D agent that also exhibits antineoplastic effects, it appears that, indeed, in diabetic patients, there is/may be a risk for developing certain types of cancer. Given the large number of altered pathways in pancreatic cancer [see diagram at http://rgd.mcw.edu/rgdweb/pathway/pathwayRecord.html?acc_id=PW:0000626, check Jones *et al.* in the reference section of the page, or see 30] and the relatively obscure etiology of T2D, determining the ‘intersection’ of the two is likely to be a daunting, albeit important task. Intriguingly, like the dedifferentiating beta cells, the pancreatic acinar cell—the most abundant cell type and possibly the cell of origin of pancreatic neoplasms—can, under certain conditions, acquire plastic capabilities, reverting to a progenitor-like state [in 30, reference 45].

Likely, coding and non-coding elements and variants within either or both, along with the machineries that control transcription and splicing, timely and tissue-specific responses and adjustments, and the factors that shape microbiota, are highly intertwined. While tight interconnectedness can confer robustness to the system as a whole, it can also promote its failure if perturbations, perhaps at strategic, or more vulnerable or in some fashion key points, combine in a reverberating, and unfortunately, ill-fated mode. And it may be, that the versatility of the endocrine/exocrine dual-function pancreas, bestows upon the specific pancreatic cells and elements within, uniquely and highly sensitive qualities that translate into varied and far-reaching responses, some certainly desired, but some unwanted, like the diabetic and neoplasmic conditions. It would be interesting to see what outcomes, the targeted and simultaneous introduction of perturbations may produce; animal models would be useful for such an approach.

A large scale meta-analysis of genome-wide association studies (GWAS) along with 1000 Genomes (1000G) imputation identifies four new loci, two each for body mass index (BMI) and fasting glucose (FG), both glycemic and obesity-related traits, and new variants of established loci. Of the four new loci, the two BMI—*ATP2B1* and *AKAP6* and one FG locus—*RMST*, derive from sex-combined analyses, while the second FG locus—*COL26A1* (known as *EMID2*), is female-specific [68]. The risk of T1D is higher if the father is affected whereas for T2D, the risk is higher if the mother is affected [1]. Interestingly, in the Non-Obese Diabetic (NOD) mouse model of T1D, the gender bias on the involvement of gut microbiota tips on the female side [6]. While the results from human and animal models appear to suggest differences in the gender bias, understanding the role it plays in the etiology and development of diabetic conditions is definitely worth pursuing.

It may be tempting to continue the journey or start a new one. Perhaps check for gender differences in the values of measurements found in PhenoMiner records, use the four newly identified loci in the imputation-based approach mentioned above, the other genes from the OLGA list (the Results Set in Fig. 8) or the genes downstream of *TCF7L2*, and design the strategy of a new expedition.

Or maybe, at this point, let the interested user design her/his own journey across the rich data landscape available at RGD, aided by tools and resources the database offers, following the leads and routes that curiosity and imagination could spark and, anticipating the wonders one might encounter, journeying here through the magnifying glass.

### 4.2 RGD

A journey through the data RGD offers was initiated using diabetes as a case study and following a selected route. Other routes could have been selected, as mentioned above. Other case study choices could have been made, as the rat has been extensively used as a model for a range of conditions. The continuous deployment of new disease portals and of pathway diagram pages, pathway suites and suite networks within the Pathway Portal are important aspects of RGD. The manual disease and pathway annotations for the human, rat and mouse genes—a distinguishing feature of RGD curation, also provide a means for targeting the rat genes associated with diseases and/or pathways for functional annotation using the three vocabularies of GO. Updates are made at the level of portals and of individual genes. A recently updated pathway suite network was mentioned in 3.3. Also relevant to the topic here is the comprehensive update on GLUT4 mediated glucose transport, both the pathway and elements within. Information on new rat strains is deposited in RGD, and more recently on sequenced strains. Data on new rat QTLs is made available, as the articles on them are published. Pipelines are developed to bring in data generated by others. Recent examples include the pipelines for OMIM, ClinVar and miRGate data import. These and the other RGD pipelines are run on a weekly basis. New pipelines for importing pathway data generated by other groups are under development. Tools are developed to access and manipulate data in various ways; OLGA is an example of a recently deployed tool. The biological knowledge is still incomplete and fragmented, the elements of the biological world are highly interconnected—these realizations only grow with every new discovery. Even for something like diabetes for which so much research has been devoted, we are far from fully understating its etiology. However, as new aspects are uncovered and others could be thought of, having large repositories of sets of biological data types and data available on them can aid in the investigation of particular issues. It can also prompt asking new questions and seeking out new venues for finding answers. RGD actively seeks to provide a comprehensive knowledge platform whose content and means to access it can offer a rewarding exploratory journey to its users.

A new disease portal – Aging & Age-Related Disease Portal, has been recently released. The miRNA gene report pages now provide Cytoscape format displays showing relationships between validated miRNA target genes and disease or pathway groups to which they are annotated.

## Conflict of Interest

The authors declare that they have no conflict of interest.
